# An *in vivo* examination of the differences between rapid cardiovascular collapse and prolonged hypotension induced by snake venom

**DOI:** 10.1038/s41598-019-56643-0

**Published:** 2019-12-27

**Authors:** Rahini Kakumanu, Barbara K. Kemp-Harper, Anjana Silva, Sanjaya Kuruppu, Geoffrey K. Isbister, Wayne C. Hodgson

**Affiliations:** 1 Monash Venom Group, Department of Pharmacology, Faculty of Medicine, Nursing and Health Sciences, Monash University, Clayton, Victoria 3168 Australia; 2 Faculty of Medicine and Allied Sciences, Rajarata University of Sri Lanka, Saliyapura, 50008 Sri Lanka; 3 Department of Biochemistry & Molecular Biology, Faculty of Medicine, Nursing and Health Sciences, Monash University, Clayton, Victoria 3168 Australia; 4 Clinical Toxicology Research Group, University of Newcastle, Callaghan, NSW 2308 Australia

**Keywords:** Cardiovascular biology, Experimental models of disease

## Abstract

We investigated the cardiovascular effects of venoms from seven medically important species of snakes: Australian Eastern Brown snake (*Pseudonaja textilis*), Sri Lankan Russell’s viper (*Daboia russelii*), Javanese Russell’s viper (*D. siamensis*), Gaboon viper (*Bitis gabonica*), Uracoan rattlesnake (*Crotalus vegrandis*), Carpet viper (*Echis ocellatus*) and Puff adder (*Bitis arietans*), and identified two distinct patterns of effects: i.e. rapid cardiovascular collapse and prolonged hypotension. *P. textilis* (5 µg/kg, i.v.) and *E. ocellatus* (50 µg/kg, i.v.) venoms induced rapid (i.e. within 2 min) cardiovascular collapse in anaesthetised rats. *P. textilis* (20 mg/kg, i.m.) caused collapse within 10 min. *D. russelii* (100 µg/kg, i.v.) and *D. siamensis* (100 µg/kg, i.v.) venoms caused ‘prolonged hypotension’, characterised by a persistent decrease in blood pressure with recovery. *D. russelii* venom (50 mg/kg and 100 mg/kg, i.m.) also caused prolonged hypotension. A priming dose of *P. textilis* venom (2 µg/kg, i.v.) prevented collapse by *E. ocellatus* venom (50 µg/kg, i.v.), but had no significant effect on subsequent addition *of D. russelii* venom (1 mg/kg, i.v). Two priming doses (1 µg/kg, i.v.) of *E. ocellatus* venom prevented collapse by *E. ocellatus* venom (50 µg/kg, i.v.). *B. gabonica*, *C. vegrandis* and *B. arietans* (all at 200 µg/kg, i.v.) induced mild transient hypotension. Artificial respiration prevented *D. russelii* venom induced prolonged hypotension but not rapid cardiovascular collapse from *E. ocellatus* venom. *D. russelii* venom (0.001–1 μg/ml) caused concentration-dependent relaxation (EC_50_ = 82.2 ± 15.3 ng/ml, R_max_ = 91 ± 1%) in pre-contracted mesenteric arteries. In contrast, *E. ocellatus* venom (1 µg/ml) only produced a maximum relaxant effect of 27 ± 14%, suggesting that rapid cardiovascular collapse is unlikely to be due to peripheral vasodilation. The prevention of rapid cardiovascular collapse, by ‘priming’ doses of venom, supports a role for depletable endogenous mediators in this phenomenon.

## Introduction

Snake venoms act as a defence against predators, aid in the capture and paralysis of prey, and assist in the digestion of prey^1^. They contain a multitude of toxins with a wide range of activities that target vital physiological processes. Many of the toxins responsible for the clinical manifestations of envenoming in humans have been extensively studied and pharmacologically/biochemically characterised. These venom components include neurotoxins^2–4^, myotoxins^5–7^, and components with pro-coagulant, anticoagulant, haemolytic and local tissue necrotic activity^8–10^. However, the nature and activity of the toxins affecting the cardiovascular system are less well understood.

There are a number of cardiovascular effects associated with snake envenoming, including hypotension, myocardial infarction, cardiac arrest, hypertension, brady- or tachy-cardia and atrial fibrillation^10–13^. Identifying the mechanism(s) responsible for venom-induced cardiovascular collapse has garnered more interest in recent years. We have previously defined ‘cardiovascular collapse’ as a sudden drop in recordable blood pressure^14^ following the administration of venom, to a laboratory animal or after human envenoming. The most common snakes responsible for this phenomenon are the brown snakes (*Pseudonaja* spp.)^15^ and, less commonly, taipans (*Oxyuranus* spp.)^14^ and tiger snakes (*Notechis* spp.)^16^. In some cases, patients spontaneously recover after collapse or respond well to basic and advanced life support^17,18^. In some cases of envenoming, particularly by brown snakes *(Pseudonaja spp.)*, the collapse can be fatal^16,17^. Indeed, in Australia, cardiovascular collapse is the leading cause of death due to snake envenoming^19^.

A number of hypotheses have been proposed to explain the cause of the cardiovascular collapse associated with snake envenoming. Previous studies have postulated that cardiovascular collapse may be due to prothrombin activators or pro-coagulant toxins present in snake venoms^20,21^. We have recently demonstrated that *in vivo* cardiovascular collapse can be caused by death adder (*Acanthophis rugosus*) venom, despite a lack of pro-coagulants in this venom. This suggests that pro-coagulant toxins are not required to induce collapse^15^. Furthermore, administering small ‘priming’ doses of *A. rugosus* venom, prior to *P. textilis* venom, prevented subsequent cardiovascular collapse. This indicated that the release of depletable endogenous mediators most likely contribute to cardiovascular collapse. We also showed that the protective effect of priming doses of venom is transient (i.e. lasting up to approximately 1 hour), indicating replenishment of mediators^15^. This suggests that clotting factors are not directly involved in cardiovascular collapse, given the longer time period required for their resynthesis. Commercial polyvalent antivenom demonstrated a protective effect on cardiovascular collapse *in vivo*, supporting a role for antigenic venom components in cardiovascular collapse^15^.

To further investigate this phenomenon, in the present study we examined the cardiovascular activity of seven medically important snake venoms: Australian Eastern Brown snake (*Pseudonaja textilis*), Sri Lankan Russell’s viper (*Daboia russelii*), Javanese Russell’s viper (*D. siamensis*), Gaboon viper (*Bitis gabonica*), Uracoan rattlesnake (*Crotalus vegrandis*), Carpet viper (*Echis ocellatus*) and Puff adder (*Bitis arietans*). We identified the species which caused cardiovascular collapse *in vivo* to further investigate the possible mechanisms for this phenomenon.

## Results

### *In vivo* experiments

For these experiments 200 µg/kg (i.v.) was chosen as a standard dose for all venoms, unless a lower dose caused a similar response (i.e. *D. siamensis* 100 µg/kg, i.v.; *E. ocellatus* 50 µg/kg, i.v.; *P. textilis* 5 µg/kg, i.v.).

The mean blood pressure and heart rate of rats prior to administration of venoms were 97 ± 16 mmHg and 255 ± 63 b.p.m., respectively.

**Figure 1:**
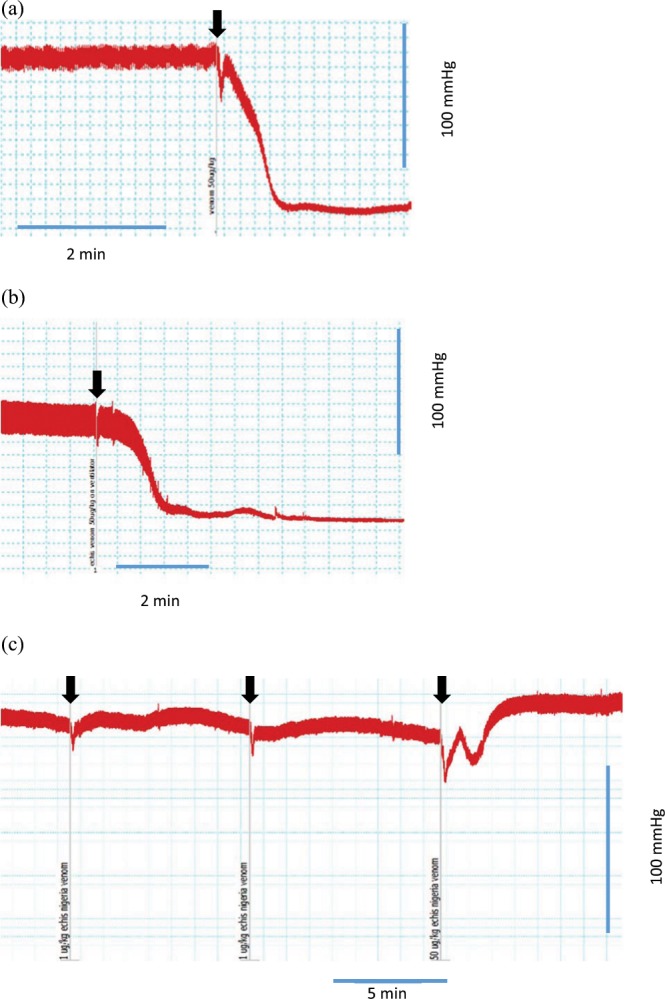
Traces showing rapid cardiovascular collapse induced by *E. ocellatus* venom (50 µg/kg, i.v.) in anaesthetised rats in the (**a**) absence and (**b**) presence of artificial respiration. **(c) **Trace showing the response to *E. ocellatus* venom (50 µg/kg, i.v.) after two sequential priming doses of *E. ocellatus* venom (1 µg/kg, i.v). Venom additions indicated by arrows.

*B. gabonica* (200 µg/kg, i.v.), *B. arietans* (200 µg/kg, i.v.), *C. vegrandis* (200 µg/kg, i.v.) and *D. siamensis* (100 µg/kg, i.v.) venoms caused relatively minor hypotensive responses (i.e. between 11 to 35% decrease) in anaesthetised rats (Table 1). *D. russelii* (100 µg/kg, i.v) caused prolonged hypotension (45 ± 8% decrease) (Table 1). *P. textilis* (5 µg/kg, i.v.) and *E. ocellatus* (50 µg/kg, i.v.) venoms induced rapid cardiovascular collapse within 2 min of venom administration (Fig. 1a; Table 1).

**Table 1:** Summary of the effects and activity of snake venoms (n = 3–6).

Species (scientific name)	Species (common name)	Dose (µg/kg, i.v.)	Maximum decrease in MAP^*^ (%)	Classified as ‘rapid cardiovascular collapse’	PLA_2_ activity (nmol/min/ml)	Procoagulant activity (Log EC_50_) (ng/ml)
*D. russelii*	Sri Lankan Russell’s viper	100	45 ± 8	No	1,334 ± 105	3.64 ± 0.12
*D. siamensis*	Javanese Russell’s viper	100	35 ± 7	No	10,237 ± 1084	3.09 ± 0.05
*B. arietans*	Puff adder	200	17 ± 2	No	378 ± 46	N/A
*C. vegrandis*	Uracoan rattlesnake	200	11 ± 1	No	1,077 ± 38	4.75 ± 0.04
*B. gabonica*	Gaboon viper	200	23 ± 3	No	3,498 ± 354	N/A
*E. ocellatus*	Carpet viper	50	100	Yes	111 ± 8	3.26 ± 0.06
*P. textilis*	Brown snake	5–10	100	Yes	473 ± 3	1.29 ± 0.05

*Within 10 min of injection.

MAP, mean arterial pressure; PLA_2_, phospholipase A_2._

**Figure 2:**
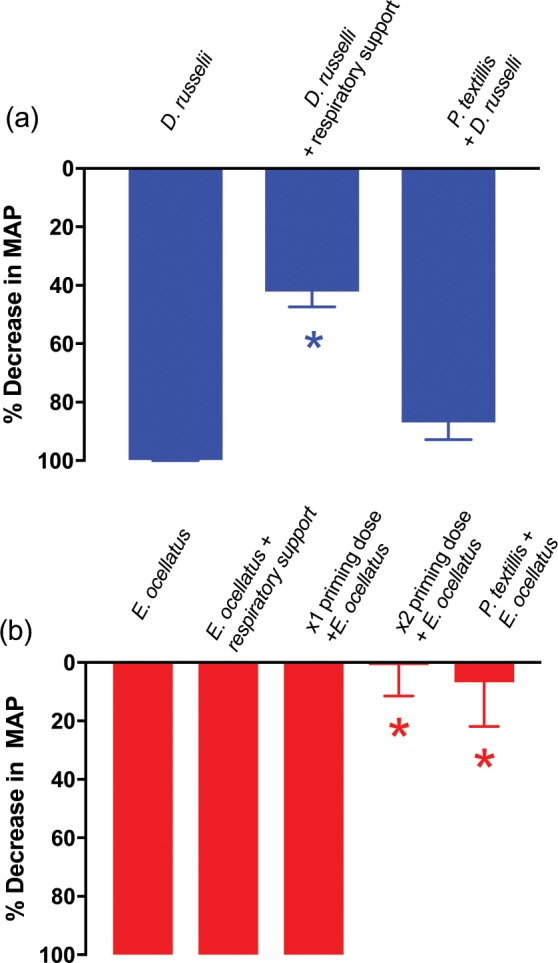
(**a**) The effects of *D. russelii* (1 mg/kg, i.v.) venom on the mean arterial blood pressure (MAP) of anesthetised rats in the presence (n = 5) or absence (n = 4) of artificial respiration, and in the presence of prior ‘priming’ with *P. textilis* venom (2 µg/kg, i.v., n = 6). (**b**) The effects of *E. ocellatus* (50 µg/kg, i.v.) venom on MAP of anesthetised rats in the presence (n = 5) or absence (n = 4) of artificial respiration, and in the presence of prior ‘priming’ with either *P. textilis* venom (2 µg/kg, i.v., n = 5), or one or two sequential doses of *E. ocellatus* venom (1 µg/kg, i.v., n = 3–4) venom. *P < 0.05 significantly different from response to same venom alone.

To investigate the effects of artificial respiratory support, a higher dose of *D. russelii* venom (1 mg/kg, i.v.) was used, which caused a 100% decrease in blood pressure. This hypotensive effect (i.e. 100%) of *D. russelii* venom (1 mg/kg, i.v.) was significantly attenuated by artificial respiratory support, reducing the hypotensive effect to 42% (Fig. 2a). In contrast, the rapid cardiovascular collapse induced by *E. ocellatus* venom (50 µg/kg, i.v.) was not attenuated by artificial respiratory support (Figs. 1b and 2b).

To explore the effect of priming doses on both types of hypotensive responses, low dose *P. textilis* venom (2 µg/kg, i.v.) was administered 10 min prior to venom administration. A priming dose of *P. textilis* venom (i.e. 2 µg/kg, i.v.) had no significant effect on the subsequent addition *of D. russelii* venom (1 mg/kg, i.v.; Fig. 2a). In contrast, a priming dose of *P. textilis* venom (2 µg/kg, i.v.) prevented rapid cardiovascular collapse induced by *E. ocellatus* venom (50 µg/kg, i.v.; Fig. 2b), as did two sequential priming doses, but not one, of *E. ocellatus* venom (1 µg/kg, i.v.; Figs. 1c and 2b).

To further investigate the above effects of the venoms, a representative venom that caused collapse (i.e. *P. textilis*) and a representative venom that caused hypotension (i.e. *D. russelii*) were injected intramuscularly. Venom doses were increased to better mimic a bite scenario. *P. textilis* venom (20 mg/kg, i.m.; Table 2) caused collapse within 10 min of administration to the left bicep femoris muscle. *D. russelii* venom (50 mg/kg or 100 mg/kg, i.m.; Table 2) caused hypotension, but not collapse, within 30 min of administration.

**Table 2:** Summary of the effects of venom (n = 4) on mean arterial blood pressure following i.m. administration.

Species (scientific name)	Species (common name)	Dose (mg/kg, i.m.)	Maximum decrease in MAP (%)	Classified as ‘rapid cardiovascular collapse’
*D. russelii*	Sri Lankan Russell’s viper	50 100	27 ± 13 52 ± 9	No
*P. textilis*	Brown snake	20	100	Yes

### PLA_2_ assay

All venoms had PLA_2_ activity. *D. siamensis* venom had the highest PLA_2_ activity, followed by *B. gabonica, D. russelii* and *C. vegrandis* venoms*. P. textilis, B. arietans* and *E. ocellatus* venoms had low PLA_2_ activity (Table 1).

### Pro-coagulant assay

**Figure 3:**
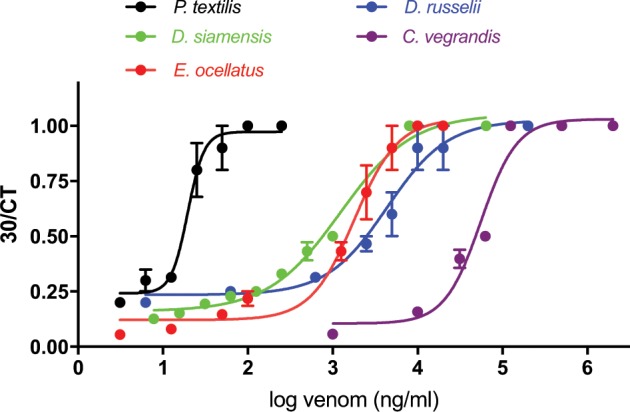
The pro-coagulant effects of venoms on the clotting time of fresh frozen plasma (n = 5–6).

*P. textilis* venom had the most potent pro-coagulant activity (i.e. logEC_50_ = 1.29 ± 0.05 ng/ml; Fig. 3; Table 1), followed by *D. russelii, D. siamensis* and *E. ocellatus* venoms. *C. vegrandis* venom (logEC_50_ = 4.75 ± 0.04 ng/ml) had less pro-coagulant activity, and *B. arietans* and *B. gabonica* venoms had no detectable pro-coagulant activity (Fig. 3; Table 1).

### *In vitro* myography experiments

**Figure 4:**
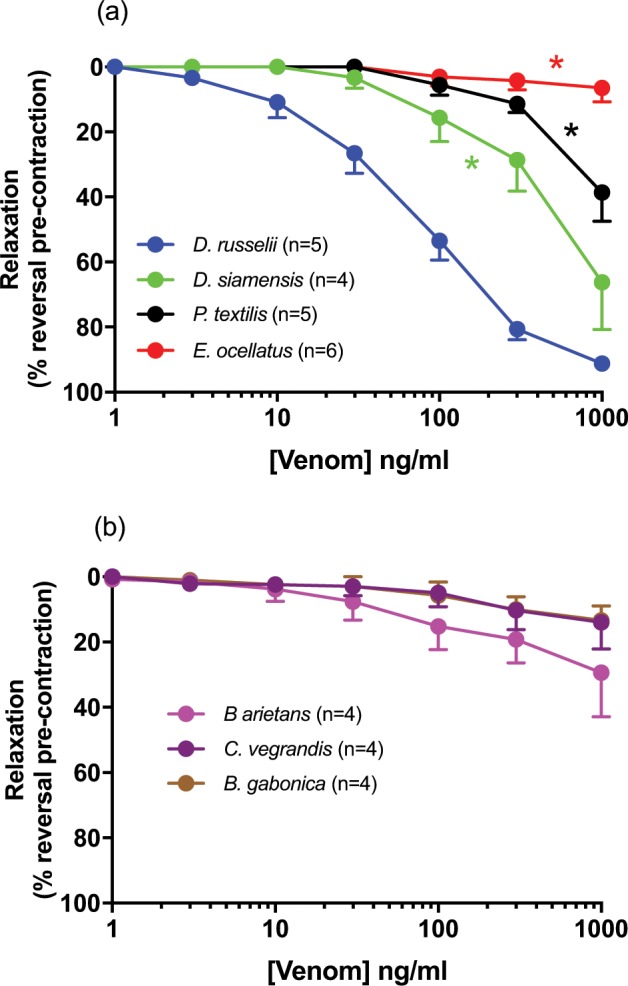
Cumulative concentration-response curves to venom (1 ng/ml − 1 µg/ml, n = 4–6) in rat small mesenteric arteries. Values are expressed as % reversal of pre-contraction and given as mean ± SEM, where *n* = number of animals. *P < 0.05, concentration-response curve significantly different as compared to *D. russelii*.

*D. russelii* venom (1–1000 ng/ml) was a potent vasorelaxant (EC_50_ = 82.2 ± 15.3 ng/ml, R_max_ = 91 ± 1%; Fig. 4a) in small mesenteric arteries. *D. siamensis* venom was a less potent vasodilator than *D. russelii* venom with an EC_50_ value of ~700 ng/ml and a relaxation response at 1000 ng/ml of 66 ± 15%. *P. textilis* venom caused < 50% relaxation (38.6 ± 9%) whilst, *E. ocellatus, B. arientans, B. gabonica and C. vegrandis* venoms induced < 30% relaxation (Fig. 4).

## Discussion

We have demonstrated two distinct patterns of cardiovascular effects caused by the intravenous administration of different snake venoms. The first group of venoms cause a rapid decrease in blood pressure, often without recovery. We refer to this as ‘rapid cardiovascular collapse’ and it is the same phenomenon that we have previously described with Australian elapid venom^15^. A defining feature of this hypotensive response is that it is attenuated by sub-toxic ‘priming’ doses of venom of the same, or different snake species^15^. Snake venoms reported to induce this effect include *P. textilis* and *E. ocellatus* in this study, and previously, *O. scutellatus* (Coastal taipan)^14^. The second group of venoms, which include *D. russelii* and *D. siamensis*, caused a slower and prolonged decrease in blood pressure, with recovery occurring in most cases. In contrast to the first group, the drop in blood pressure is not prevented by prior administration of priming doses. We refer to this effect as ‘prolonged hypotension’.

We have previously postulated that the attenuation of the hypotensive effect with prior administration of smaller sub-toxic doses of venom is due to the pre-release, and depletion, of mediators which induce collapse^15^. This phenomenon was observed in the current study in which smaller priming doses of *E. ocellatus* venom or *P. textilis* venom prevented cardiovascular collapse caused by a larger dose of *E. ocellatus* venom. This suggests that these venoms are inducing their cardiovascular effects via a common mechanism.

For a high dose of *D. russelii* venom (i.e. 1 mg/kg), a response similar to rapid cardiovascular collapse occurred. However, when the rat was placed on a ventilator prior to administration of venom, this so called ‘collapse’ was prevented. In contrast, when rats administered *E. ocellatus* venom were placed on the ventilator, rapid cardiovascular collapse still occurred. The reasons for the protective effects of supportive respiration are unclear. We have previously shown that the neurotoxins present in *D. russelii* venom are relatively weak^22^. However, given that a rat has approximately 64 ml of circulating blood per kg body weight, an intravenous dose of 1 mg/kg of venom leads to a blood concentration of approximately 16 µg/ml. This very high venom concentration may be sufficient to cause paralysis of the diaphragm given that a 30 ng/ml concentration of the same venom caused complete neuromuscular blockade in the chick biventer nerve-muscle preparation^22^. Therefore, it could be argued that artificial respiration is preventing or overcoming the paralytic effects of the neurotoxins on the rat diaphragm. The different effects of supportive respiration on the cardiovascular effects of the venoms also supports the fact that collapse due to *E. ocellatus* venom occurs via a different mechanism. These studies were conducted *in vivo* using ketamine/xylazine as anesthesia, which may have affected the blood pressure, although ketamine is more likely to cause a slight increase in blood pressure.

To ensure that these cardiovascular effects seen in the *in vivo* model occurs in an actual snake bite, the effects of *P. textilis* venom and *D. russelii* venom were also tested via intramuscular administration. At 20 mg/kg (i.m.), *P. textilis* venom caused collapse within 10 min of administration. This delay in response is likely to be due to the time it takes for the venom to be absorbed. In contrast, when *D. russelii* venom was administered via intramuscular injection prolonged hypotension occurred, similar to that observed when venom was administrated intravenously. Even at 100 mg/kg concentration, collapse did not occur, further highlighting that both collapse and hypotension are not dose-dependent responses but represent two distinct cardiovascular effects.

There are many factors that could lead to venom-induced hypotension^11^, as distinct from cardiovascular collapse. Some snake venoms have highly evolved toxins such as calciseptine, FS2 toxins, C10S2C2 and S4C8 which block L-type Ca^2+^ currents^23,24^. Increasing capillary permeability protein (ICPP), isolated from Blunt-nosed viper *(V. lebtina)* venom is similar in potency and structure to vascular endothelial growth factor (VEGF) and is responsible for increasing vascular permeability^25^. Natriuretic peptides found in Green Mamba *(D. angusticeps)* venom^26^ and bradykinin potentiating peptides found in *Bothrops spp*. are also potent vaso-relaxants^10,27,28^. In the current study, *D. russelii* venom caused concentration-dependent relaxation of rat small mesenteric arteries suggesting peripheral vasodilation contributes to the prolonged hypotension observed *in vivo*. *D. siamensis* venom was also an efficacious dilator of rat small mesnteric arteries, though less potent than *D. russelii* venom. In contrast, the venoms which had a modest hypotensive effect *in vivo* (*B. arientans, C. vegrandis and B. gabonica*) were poor vasorelaxants of isolated mesenteric arteries. Although vasorelaxant responses can exhibit heterogeneity throughout the vasculature, the mesenteric vascular bed was chosen for this study given it makes a significant contribution to overall total peripheral resistance, receiving 25% of total cardiac output. As such, characterising vasorelaxation responses in these small mesenteric arteries (approx. 300microns in diameter), is of physiological relevance to blood pressure control. Gaboon viper *(B. gabonica)* venom has been shown to induce vasodilation resulting in a drop in peripheral resistance, leading to reduction in stroke volume due to cardiotoxins^29^. In another study using isolated heart preparations, Rhinoceros viper *(B. nasicornis)* venom produced an increase in left ventricular pressure, pacemaker activity and heart rate, indicating that the venom contains toxins that disrupt [Ca^2+^] and ion conductance^30^.

PLA_2_ toxins are ubiquitous components of snake venoms and display an array of activities including neurotoxicity, myotoxicity, cardiotoxicity, anti-coagulation, haemolytic, hypotensive and local tissue necrotic activity^31^. Interestingly, *P. textilis* and *E. ocellatus* venom, which induced rapid cardiovascular collapse, had the lowest PLA_2_ activity, whereas *Daboia spp*. had the highest amount of PLA_2_ activity. Therefore, there does not seem to be a link between PLA_2_ activity  and cardiovascular collapse, although these toxins may contribute, directly or indirectly, to prolonged hypotension via vasodilation.

*Daboia spp*. and *Pseudonaja spp*. contain pro-coagulants factors in their venom^32^. While both *P. textilis* and *E. ocellatus* venoms caused rapid cardiovascular collapse *in vivo*, *P. textilis* venom was most potent in causing coagulation while *E. ocellatus* venom possessed comparatively less pro-coagulant activity. *D. russelii* and *D. siamensis* venom also caused coagulation. *D. russelii* and *D. siamensis* venoms are known to contain Factor X^33,34^ while both *E. ocellatus*^35^ and *P. textilis* venom contain prothrombin activators^21,36,37^. Therefore, pro-coagulant activity is unlikely to be directly related to the cardiovascular collapse induced by the venoms.

In conclusion, we have shown that the *in vivo* cardiovascular effects of venom include, at least, two distinct phenomena i.e. rapid cardiovascular collapse and prolonged hypotension and that both effects involve different mechanisms. Rapid cardiovascular collapse has a sudden onset and appears to be mediated by depletable endogenous mediators. In contrast, prolonged hypotension has a slower onset and appears to be due mainly to vasodilation.

## Methods

### Materials

Drugs and materials used were ketamine (Ceva Animal Health, Australia), xylaxine (Troy Laboratories Pty, Ltd, Australia), heparin (Hospira, Germany), bovine serum albumin (Sigma, USA), and fresh frozen plasma (Australian Red Cross). *D. siamensis, P. textilis, B. arietans, B. gabonica* and *C. vegrandis* venoms were obtained from Venom Supplies (Australia). *D. russelii* venom was a gift from Professor A. Gnanadasan (University of Colombo). *E. ocellatus* venom was a gift  from the Liverpool School of Tropical Medicine. For procoagulant assays, venom (1 mg/mL) was prepared in 0.5% bovine serum albumin/tris-buffered saline and stored at −20 °C. Dilutions were prepared in 0.5% BSA/TBS immediately before use.

Animal experiments were approved by the Monash University Ethics Committee (MARP/2014/097 and MARP/2017/147). All experiments were performed in accordance with relevant guidelines and regulations.

### Anaesthetised rats

Male Sprague-Dawley rats (280–350 g) were anaesthetised with a mixture of ketamine (100 mg/kg, i.p.) and xylazine (10 mg/kg, i.p.). Ketamine/xylazine cocktail was used as it provides sedation and muscle relaxation as well as deep analgesia and anesthesia without compromising blood pressure. A midline incision was made and a cannula inserted into the trachea for mechanical ventilation (~1 ml/100 g of body weight at 55 strokes/min) if required. Cannulae were inserted into the left jugular vein for administration of venom and the right carotid artery to record arterial blood pressure. The arterial cannula was connected to a pressure transducer. Blood pressure was then allowed to stabilise for approximately 10–15 min. Body temperature was maintained at 37 °C using an overhead lamp and heated dissection table. Venom was administered via the jugular vein followed by flushing with saline or via a bolus administration into the left bicep femoralis muscle. Responses to venom were measured as percentage change in mean arterial pressure (MAP).

### Myograph experiments

Male Sprague-Dawley rats (200–250 g) were euthanized by CO_2_ inhalation (95% CO_2_, 5% O_2_) followed by cervical dislocation. Small mesenteric arteries (third-order branch of the superior mesenteric artery) were isolated, cut into 2 mm lengths, and mounted in isometric myograph baths. Vessels were maintained in physiological salt solution, composed of (in mM): 119 NaCl, 4.7 KCl, 1.17 MgSO_4_, 25 NaHCO_3_, 1.8 KH_2_PO_4_, 2.5 CaCl_2_, 11 glucose, and 0.026 EDTA, at 37 °C and supplied with carbogen (95% O_2_; 5% CO_2_). The mesenteric arteries were allowed to equilibrate for 30 min under zero force and then a 5 mN resting tension was applied. Changes in isometric tension were recorded using Myography Interface Model 610 M version 2.2 (ADInstruments, Pty Ltd, USA) and a chart recorder (Yokogawa, Japan). Following a 15 min equilibration period at 5 mN, the mesenteric arteries were contracted maximally (F_max_) using a K^+^ depolarizing solution [K^+^-containing physiological salt solution (KPSS); composed of (in mM) 123 KCl, 1.17 MgSO_4_, 1.18 KH_2_PO_4_, 2.5 CaCl_2_, 25 NaHCO_3_, and 11 glucose]. The integrity of the endothelium was confirmed by relaxation to acetylcholine (ACh, 10 µM) in tissues pre-contracted with the thromboxane A_2_ mimetic, U46619 (1 µM), then washed with physiological salt solution and the tension allowed to return to baseline. Relaxation of >80% to ACh was used to indicate vessels with an intact endothelium. There were no significant differences in response to ACh between the groups studied. If endothelial damage was evident (ACh relaxation <80%) then the vessel was not used for experimentation. Cumulative concentration-response curves to venom (1 ng/ml-1µg/ml) were constructed in vessels pre-contracted with titrated concentrations of U46619 (~50% F_max_). Sodium nitroprusside (SNP, 10 µM) was added at the end of each concentration-response curve to ensure maximum relaxation. Only one concentration-response curve to venom was obtained in each vessel segment^38,39^.

### Pro-coagulation assay

Aliquots (10 ml) of fresh frozen plasma were thawed at 37 °C, then spun at 2500 rpm for 10 min. Venom solutions (100 µL) were placed in the wells of a 96 well microtitre plate at room temperature or at 37 °C in a BioTek ELx808 plate reader. Plasma (100 µL) and calcium (0.2 M/ml) were then added simultaneously to each well using a multichannel pipette. After a 5 s shake step for mixing, the optical density at 340 nm was monitored every 30 s over 20 min^40^.

### PLA_2_ assay

PLA_2_ activity of the venoms was determined using a secretory PLA_2_ colourmetric assay kit (Cayman Chemical; MI, USA) according to manufacturer’s instructions. This assay used 1, 2-dithio-analogue of diheptanoyl phosphatidylcholine, which serves as a substrate for PLA_2_ enzymes. Free thiols generated following the hydrolysis of the thioester bond at the *sn*-2 position by PLA_2_ are detected using DTNB (5, 5′-dithio-bis-[2-nitrobenzoic acid]). Colour changes were monitored at 405 nm in a fusion α microplate reader (PerkinElmer; MA, USA), sampling every minute for a 5 min period. PLA_2_ activity was expressed as micromoles of phosphatidylcholine hydrolysed per minute per milligram of enzyme^2^.

### Statistical analysis

For the anaesthetized rat experiments, pulse pressure was defined as the difference between systolic and diastolic blood pressures. Mean arterial pressure (MAP) was calculated as diastolic blood pressure plus one-third of pulse pressure. These data were tested using a D’Agostino-Pearson normality test and found to be normally distributed. Therefore, differences in MAP between treatment groups were analysed using a one-way ANOVA with Dunnett’s multiple comparison test. Sample sizes are based on the number of animals required to provide >85% power to detect an effect size of 35% with a confidence level (α) of 5% for the *in vivo* endpoint measure of blood pressure (standard deviation (SD) <15%). This ensured that experimental design was sufficiently powered.

For the myography experiments, blood vessel relaxation was expressed as a percentage reversal of the U46619 pre-contraction. Individual relaxation curves to *D. russelii* venom were fitted to a sigmoidal logistic equation and EC_50_ values (concentration of agonist resulting in a 50% relaxation) calculated^41^. Where EC_50_ values could not be obtained, concentration-response curves to venoms were compared by means of a two-way repeated measures ANOVA (n = number of artery segments from separate animals). Data represent the mean ± SEM (error bars on graph). Statistical significance was defined as P < 0.05. All data analysis was performed using GraphPad Prism version 5.02 (GraphPad Software, San Diego, CA, USA).

For the coagulation assay, responses were plotted as 30 s/[clotting time(s)] against the logarithm of the venom concentration. This provided a normalised measure of the clotting effect and produced normalised concentration-clotting curves, which were fitted with a standard sigmoidal curve (Hill slope = 1) to calculate the effective concentration 50 (EC_50_). The EC_50_ is the concentration of venom that resulted in a pro-coagulant effect halfway between no clotting effect and maximal clotting effect^42^.
